# Discharge teaching quality positively predicts quality of life in colorectal cancer patients with temporary enterostomy: The mediating role of readiness for hospital discharge and stoma self-efficacy

**DOI:** 10.1371/journal.pone.0306981

**Published:** 2024-07-11

**Authors:** Liying Lin, Yifang Fang, Feifei Huang, Xiaoying Zhang, Jianwei Zheng, Huimin Xiao

**Affiliations:** 1 Department of Gastrointestinal Surgery, The First Affiliated Hospital of Fujian Medical University, Fuzhou, China; 2 Department of Colorectal Surgery, The First Affiliated Hospital of Fujian Medical University, Fuzhou, China; 3 School of Nursing, Fujian Medical University, Fuzhou, China; 4 Department of Oncology, The Union Hospital Affiliated with Fujian Medical University, Fuzhou, China; 5 Research Center for Nursing Humanity, Fujian Medical University, Fuzhou, China; E-Da Cancer Hospital, TAIWAN

## Abstract

**Objectives:**

This study aimed to examine the mediating role of readiness for hospital discharge (RHD) and stoma self-efficacy (SSE) in the relationship between quality of discharge teaching (QDT) and health-related quality of life (HRQOL) in colorectal cancer patients with temporary enterostomy, and the gender difference of mediating effect.

**Background:**

It is not clear how RHD, QDT, SSE and HRQOL interact in colorectal cancer patients with temporary enterostomy.

**Methods:**

This was a prospective follow-up survey. 221 colorectal cancer patients with temporary enterostomy were conveniently recruited from a general hospital in Southeast China. The Quality of Discharge Teaching Scale, Readiness for Hospital Discharge Scale, Stoma Self-Efficacy Scale, and Stoma Quality of Life Scale were used to collect data. Pearson’s correlation and structural equation models were used to analyze the data. SPSS 26.0 and Amos 28.0 software were used for analysis the collected data.

**Results:**

Regarding the relationship of QDT and HRQOL, only QDT-T had a direct effect among colorectal cancer patients with stomas (b = 0.233, *P*<0.001, percentile 95% CI = [0.145, 0.314]). However, both QDT-T and QDT-R can predict HRQOL indirectly through three paths: (1) the mediating role of SSE (b = 0.050, *P* = 0.009, percentile 95% CI = [0.013, 0.098]; b = 0.077, *P* = 0.008, percentile 95% CI = [0.021, 0.164]), (2) the mediating role of RHD (b = 0.044, *P* = 0.004, percentile 95% CI = [0.014, 0.085]; b = 0.044, *P* = 0.005, percentile 95% CI = [0.010, 0.102]), and (3) the chain mediating role of SSE and RHD (b = 0.030, *P* = 0.003, percentile 95% CI = [0.011, 0.059]; b = 0.047, *P* = 0.003, percentile 95% CI = [0.015, 0.103]). The similar chain mediating effect in male stoma patients was also found (b = 0.041, *P* = 0.002, percentile 95% CI = [0.016, 0.080]; b = 0.046, *P* = 0.004, percentile 95% CI = [0.011, 0.114]).

**Conclusions:**

Stoma self-efficacy and readiness for hospital discharge played important intermediary roles in the relationship between quality of discharge teaching and health-related quality of life in colorectal cancer patients with stomas. Health care providers can design SSE-enhancing and RHD-enhancing discharge planning for colorectal cancer patients with temporary enterostomies.

## Introduction

Colorectal cancer ranks the third most prevalent cancer globally [1]. Temporary enterostomy is one of its potentially curative surgical therapies [2]. However, it may cause various transitional problems, such as peristomal skin issues, sexual problems, and low psychosocial adaptation [3, 4]. Which strongly predict unplanned readmission, and even lead to about 10% permanent stomas [5]. Recently, a systematic review [6] found that discharge planning can effectively reduce transition risks and improve patients’ Health-related quality of life (HRQOL). It is necessary to understand how discharge planning affects HRQOL in patients with colorectal cancer undergoing temporary enterostomy.

Quality of discharge teaching (QDT) is a basic component of discharge planning, involving patient teaching content and skills [7]. It is essential for safe transitions of care [8]. QDT greatly affects patients’ post-discharge outcomes, such as complications, readmission rates, and post-discharge coping competence [9, 10]. Studies have pointed out that the QDT provided by medical staff has an important impact on the quality of life of patients with stoma and care experience of their family caregivers [11, 12]. Weiss’s studies further indicated that readiness for hospital discharge (RHD) plays an important intermediary role between QDT and post‑discharge outcomes in surgical patients and mothers after birth [13, 14]. The similar mechanism needs to be verified in colorectal cancer patients with stomas.

RHD is an indicator used to evaluate discharge planning, focusing on whether patients complete a safe discharge process, continue further rehabilitation, and return to normal societal functioning [15]. With enhanced recovery pathways post-surgery, the average length of hospitalization for patients with stomas has shortened significantly, resulting in insufficient RHD [16]. Zhao et al. [17] reported a positive correlation between RHD and QDT in colorectal cancer patients post-colostomy. Additionally, lower RHD was founded to co-occur with lower HRQOL in these patients [18].

Stoma self-efficacy (SSE) reflects patients’ conviction that they can successfully manage their stomas to minimize adverse outcomes. It is identified to be positively correlated with HRQOL in patients with a stoma, whether permanent or temporary [5, 11]. Several studies have indicated that SSE and QDT are both positively correlated with RHD in stoma patients [19, 20]. Zhang [9] have further revealed that QDT-T has a positive impact on RHD, whereas QDT-R was not.

**Fig 1:**
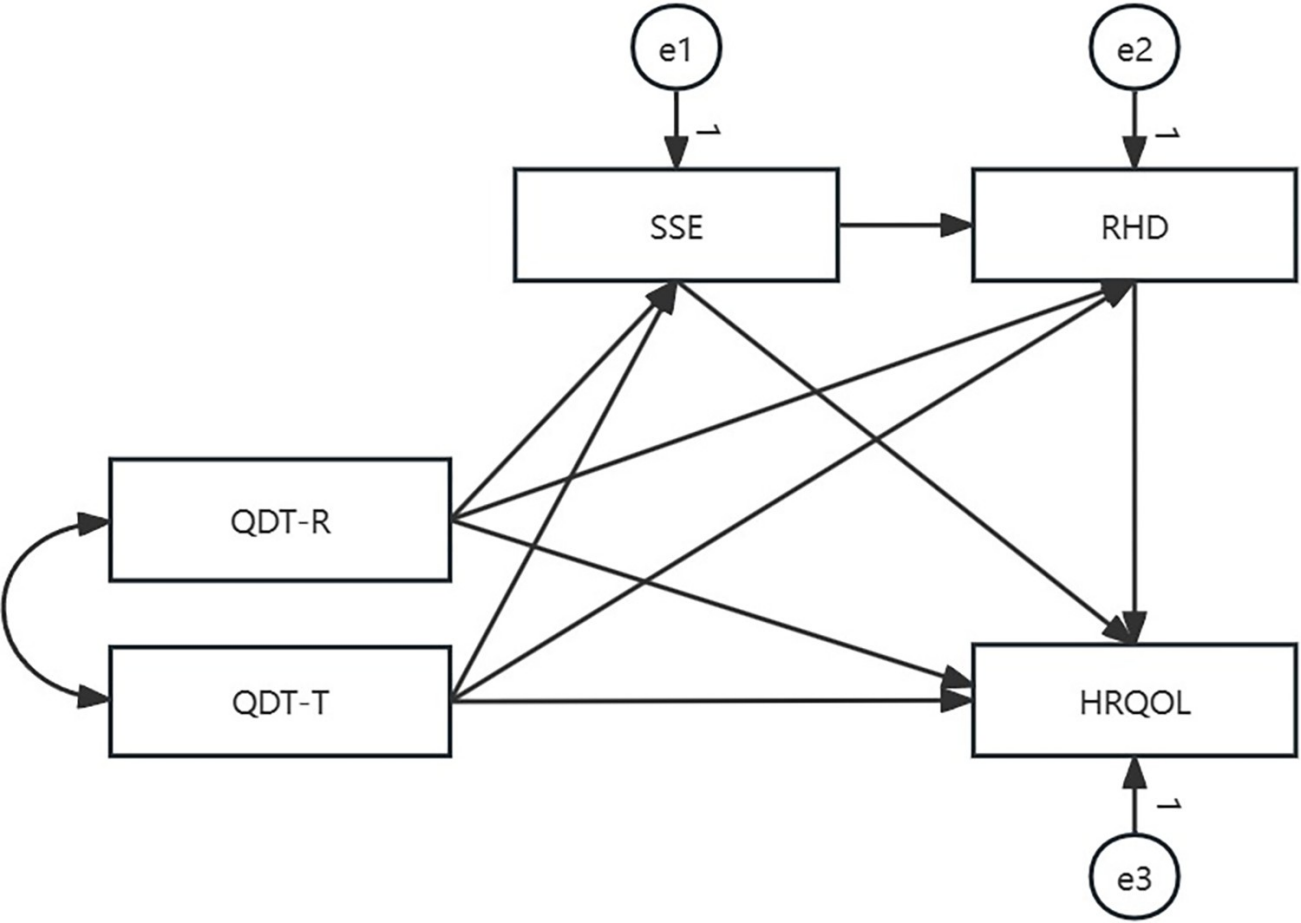
Hypothetical path model with all colorectal cancer patients with temporary enterostomy (*n* = 221).

Guided by Meleis’ transitions theory model and literature review, it was hypothesized that QDT-R and QDT-T have a direct effect on HRQOL and an indirect effect on HRQOL mediated by SSE and RHD (Fig 1). This theory explains how transition types, transition conditions, and nursing therapeutics affect the process or outcome indicators. Colorectal cancer patients experience a transition from the hospital to home after a temporary enterostomy. QDT as a nursing therapy can help patients to regain stability and a sense of well-being. SSE and RHD are process indicators during this transition. HRQOL is an outcome indicator used to evaluate the effectiveness of the transition. Given differences in HRQOL between the sexes of enterostomy patients [21, 22], one objective of this study was to determine whether this mechanism is affected by sex. Therefore, this study aimed to examine whether the relationship between QDT and HRQOL is mediated by SSE and RHD, and to identify sex-based differences in the mediating effects of SSE and RHD.

## Materials and methods

### Study design and participants

This is a prospective follow-up survey. Using maximum likelihood estimation, fit statistics can only provide appropriate conclusions about a model when the sample size is greater than 200 [23]. Thus, a total of 221 colorectal cancer patients with temporary enterostomies were conveniently recruited from April 2022 to October 2022 at a university affiliated general hospital in Fuzhou, southeast of China. The inclusion criteria were: (a) diagnosed with colorectal cancer without distant metastasis to solid organs or other tumors; (b) aged 18 years or older; and (c) undergoing their first admission for temporary enterostomy. The exclusion criteria were: (a) diagnosed with severe organic disease; (b) suffering from operation intolerance or severe complications; and (c) unable to communicate.

### Data collection

Ethical approval was obtained from the corresponding author’s university prior to this study (IRB No.2022-00069). The data for this study were collected by two trained research assistants (RA). On the day of discharge from hospital, one RA collected data using a self-reported personal information questionnaire, which utilized elements of the Quality of Discharge Teaching Scale (QDTS), the Stoma Self-efficacy Scale (SSES), and the Readiness for Hospital Discharge Scale (RHDS). At the 30-day post-discharge, the other RA conducted a telephone interview to assess the patients’ HRQOL. They also gathered data on unplanned readmissions from medical records. Finally, 225 questionnaires were distributed and 221 valid questionnaires were obtained, with an effective recovery rate of 98.2%.

### Measures

A self-reported personal information questionnaire was designed to collect sociodemographic data including sex, age, education level, marital status, work status, monthly income, residence, living status (living alone: yes/no), and health insurance. Medical variables included medical diagnosis, disease stage, surgical mode, stoma type, postoperative hospital length of stay, postoperative radiotherapy or chemotherapy (yes/no), stoma complications (yes/no), and unplanned readmission (yes/no). Data were collected from the participating patients’ medical records.

QDT was measured using the Chinese version of the QDTS [24]. This scale consists of 24 items scored from 0 to 10 (0 = none at all; 10 = very much). The scale has three dimensions: “needed content,” “received content,” and “teaching skills and effects.” The total score is the sum obtained across all three of these dimensions and ranges from 0 to 180. Higher scores represent greater satisfaction with QDT. Cronbach’s α was 0.91 for the total scale, and was 0.935, 0.882, and 0.923 for the three different dimensions, respectively.

SSE was measured using the Chinese version of the SSES [25]. The scale consists of 28 items, covering two dimensions of “stoma self-efficacy” and “stoma social efficacy”, and six individual items. Each item was evaluated from 1 (no confidence) to 5 (very confident). Thus, the total score ranges from 28 to 140, with higher scores indicating higher levels of individual adaptability. A score of less than 65 indicates low efficiency, a score between 66 and 102 indicates moderate efficiency, and any score above 103 indicates high efficiency. Cronbach’s α was 0.97 for the total scale, and 0.97 and 0.89 for the two dimensions.

The RHDS was originally developed by Weiss and Piacentine [26]. The Chinese version, validated by Lin [27] was used to assess participants’ RHD. The Chinese version of the RHDS is a 12-item scale covering three dimensions of “personal status”, “adaptability”, and “expected support”. Each item is scored on a numerical scale ranging from 0 to 10 (0 = worst, 10 = best), with total scores ranging from 0 to 120. Higher scores represent better readiness for discharge. Cronbach’s α for this scale was 0.89.

The Stoma Quality of Life Scale (SQOL) was used to measure the HRQOL of colorectal cancer patients with temporary enterostomy. It was first developed by Prieto [28] and was translated and validated into Chinese by Wu et al. [29]. The Chinese version of the SQOL contains four dimensions and 20 items. Each item is scored from 1 to 4 (1 = always, 4 = never), with higher scores indicating a higher level of satisfaction with HRQOL. Cronbach’s α for this scale was 0.893.

### Data analysis

Descriptive analyses were conducted using IBM SPSS Statistics Version 26.0. Categorical variables were reported as frequencies and percentages, and continuous variables were reported as means and standard deviations. Pearson’s correlation analysis was used to assess QDT-R, QDT-T, SSE, RHD, and HRQOL.

Path models were generated using Amos Version 28.0. Hayes’ bootstrapping method was used to examine the mediating effects of SSE and RHD on QDT and HRQOL. 5000 bootstrap samples were generated to produce bootstrap confidence interval (CI) for the indirect effect. An indirect effect was assumed significant at an alpha level of 0.05 if the 95% CI did not include zero [30].

Model fit was determined by several goodnesses of fit indexes: the values of the Chi-Square (χ2) test, degrees of freedom (df), the goodness of fit index (GFI), the adjusted goodness of fit index (AGFI), the comparative fit index (CFI), the incremental fit index (IFI), the Tucker–Lewis Index (TLI), and the root mean square error of approximation (RMSEA). The following values were used for an acceptable fit of the model: χ2/df<3. 00, GFI>0. 90, AGFI>0. 90, CFI>0. 90, IFI>0. 90, TLI>0. 90, and RMSEA<0. 08 [31].

## Results

### Participant characteristics

The average age of the 221 participants was 61.52 years, with 59.3% aged 60 years or older. More than half of the patients were male (65.2%), had received a junior high school level of education or higher (68.8%), lived in towns (56.9%), and had medical insurance for urban employees and residents (67.0%). 47.1% of the patients were unemployed and approximately 20% of the patients’ monthly income was below $208. Most participants had spouses (91.9%) and did not live alone (95.4%). In terms of medical characteristics, the vast majority of patients had been diagnosed with rectal cancer (89.6%) in stages II–IV (92.3%), had undergone a laparoscopic surgery (80.5%), and had rectal stomas (98.4%). The average length of participants’ postoperative hospital stay was 11.9 days, and 73.3% of patients received postoperative radiotherapy or chemotherapy. Within 30-days of discharge, more than half of the patients experienced stoma complications (52.0%), and 13.6% underwent unplanned readmission (Table 1).

**Table 1:** Demographic characteristics of patients (*n* = 221).

Variable	Frequency (%)
**Sex**	
Male	144(65.2%)
Female	77(34.8%)
**Age (years)**	
18–59	90(40.7%)
≥60	131(59.3%)
**Educational level**	
Primary school or below	69(31.2%)
Junior high school	90(40.7%)
Technical secondary school and junior college	53(24.0%)
Undergraduate or above	9(4.1%)
**Marital status**	
Unmarried	2(0.9%)
Married	203(91.9%)
Divorced	1(0.5%)
Widowed	15(6.8%)
**Monthly income per person (RMB)**	
<1500	44(19.9%)
1500–2500	30(13.6%)
2501–3500	70(31.7%)
>3500	77(34.8%)
**Residence**	
Town	126(57.0%)
Countryside	95(43.0%)
**Living alone**	
Yes	10(4.5%)
No	211(95.5%)
**Health insurance**	
Public expense	2(0.9%)
Own expense	1(0.5%)
New rural cooperative medical insurance	97(43.9%)
Medical insurance for urban employees/ resident	115(52.0%)
Commercial insurance	6(2.7%)
**Working status**	
On job	37(16.7%)
Retirement	80(36.2%)
Unemployed	104(47.1%)
**Medical diagnosis**	
Colon cancer	18(8.1%)
Rectal cancer	203(91.9%)
**Operation mode**	
Laparoscope	178(80.5%)
Open	43(19.5%)
**Staging of disease**	
Phase I	17(7.7%)
Phase II	80(36.2%)
Phase III	73(33.0%)
Phase IV	51(23.1%)
**Stoma type**	
Colostomy	23(10.4%)
Ileostomy	198(89.6%)
**Postoperative hospital stay (days)**	
≤ 10	105(47.5%)
≥ 11	116(52.5%)
**Postoperative radiotherapy or chemotherapy**	
Yes	162(73.3%)
No	59(26.7%)
**Complication**	
Yes	115(52.0%)
No	106(48.0%)
**Unplanned admission**	
Yes	30(13.6%)
No	191(86.4%)

### Descriptive statistics and correlations among the main variables

Table 2 shows the QDT, RHD, SSE, and HRQOL scores and their significant correlations. The scores of QDT, SSE, RHD, and HRQOL were 102.52±12.65, 59.93±9.35, 67.65**±**8.61, and 43.81±5.32 (Mean±SD), respectively. The scores of both dimensions of QDT were 33.48 ±4.72 and 69.01±8.81 (Mean±SD), respectively. The QDT-R, QDT-T, SSE, RHD, and HRQOL were all significantly correlated with each other. QDT-R had a moderately strong correlation with QDT-T, SSE, RHD, and HRQOL (r = 0.714, r = 0.665, r = 0.683, r = 0.620, *p*<0.01). QDT-T was positively correlated with SSE, RHD, and HRQOL (r = 0.687, r = 0.733, r = 0.699, *p*<0.01).

**Table 2:** Descriptive statistics and correlations among the variables (*n* = 221).

Variables	M	SD	Correlations among variables					
			1	1a	1b	2	3	4
**1 QDT**	102.52	12.65	1	0.871**	0.965**	0.729**	0.767**	0.721**
**1a QDT-R**	33.48	4.72	-	1	0.714**	0.665**	0.683**	0.620**
**1b QDT-T**	69.01	8.81	-	-	1	0.687**	0.733**	0.699**
**2 SSE**	59.93	9.35	-	-	-	1	0.779**	0.649**
**3 RHD**	67.65	8.61	-	-	-	-	1	0.679**
**4 HRQOL**	43.81	5.32	-	-	-	-	-	1

Note: QDT: Quality of Discharge Teaching; QDT-R: QDT-Received content; QDT-T: QDT-Teaching skills and effects; SSE: Stoma Self-Efficacy; RHD: Readiness for Hospital Discharge; HRQOL: Health-Related Quality of Life

**P<0.01

### Mediating effects of SSE and RHD

**Fig 2:**
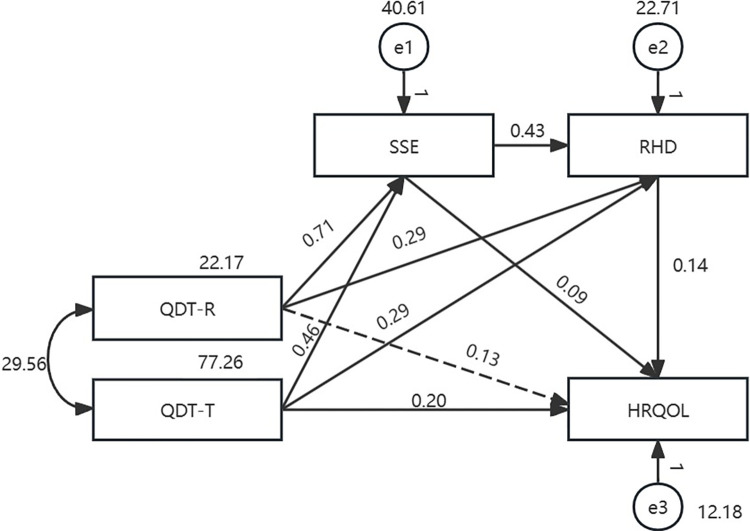
Initial path model with all colorectal cancer patients with temporary enterostomy (*n* = 221).

**Fig 3:**
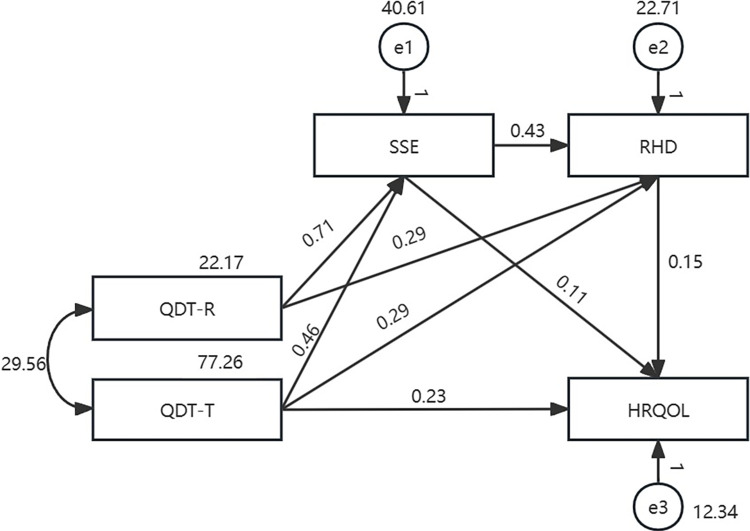
Adjusted path model with all colorectal cancer patients with temporary enterostomy (*n* = 221).

The findings on the mediating effects of SSE and RHD on the relationship between the two dimensions of QDT (QDT-R and QDT-T) and HRQOL are presented in Table 3. The path model with all colorectal cancer patients with temporary enterostomy (model Ⅰ) is presented in Fig 2. The maximum likelihood method was used to fit and modify the model. The fitting indicators were not within the acceptable range, indicating that the model fit was unacceptable. It demonstrated “QDT-R→ HRQOL” was not significant (b = 0.13, *p* = 0.093). Based on model Ⅰ, another model (model Ⅱ) was established. The fitting indicators were: χ2 = 2.808, df = 1, χ2/df = 2.808, GFI = 0.995, AGFI = 0.924, NFI = 0.996, CFI = 0.998, IFI = 0.998, TLI = 0.976, and RMSEA = 0.091, all of which were within the acceptable range. The path analysis results revealed that all paths in the proposed model were significant (*p*<0.001). The total effect between the two dimensions of QDT and HRQOL was significant (b = 0.525, *p*<0. 001, percentile 95% CI = [0.437, 0.616]). The direct effect accounted for 44.4% of the total effect (b = 0.233, *p*<0.001, percentile 95% CI = [0.145, 0.314]) and the mediating effect accounted for 55.6% of the total effect (b = 0.292, *p*<0. 001, percentile 95% CI = [0.180, 0.423]). Among the mediating effect, the chain mediation of “QDT-R→SSE → RHD→ HRQOL” and “QDT-T→SSE → RHD→ HRQOL” were both significant (b = 0.047, *p =* 0.003, percentile 95% CI = [0.015, 0.103]; b = 0.030, *p =* 0.003, percentile 95% CI = [0.011, 0.059]) (Fig 3 and Table 3).

**Table 3:** Mediating effect of SSE and RHD.

		Model Ⅱ				Model Ⅲ							
		All patients (n = 221)				Male patients (n = 144)				Female patients (n = 77)			
		Point estimate	p	Bootstrap 5000times 95%CI		Point estimate	p	Bootstrap 5000times 95%CI		Point estimate	p	Bootstrap 5000times 95%CI	
				Lower	Upper			Lower	Upper			Lower	Upper
**Total Effects**		0.525	<0.001	0.437	0.616	0.496	<0.001	0.386	0.605	0.550	<0.001	0.428	0.721
**Direct effects QDT-T → HRQOL**		0.233	<0.001	0.145	0.314	0.184	0.001	0.077	0.301	0.319	0.001	0.186	0.436
**Total Indirect effects**		0.292	<0.001	0.180	0.423	0.312	<0.001	0.174	0.468	0.231	0.009	0.058	0.484
**Indirect effects**	**QDT-R→SSE → RHD→ HRQOL**	0.047	0.003	0.015	0.103	0.046	0.004	0.011	0.114	0.023	0.309	-0.022	0.121
	**QDT-R→SSE→ HRQOL**	0.077	0.008	0.021	0.164	0.058	0.053	-0.001	0.159	0.114	0.021	0.013	0.275
	**QDT-R → RHD→ HRQOL**	0.044	0.005	0.010	0.102	0.062	0.011	0.011	0.150	0.015	0.323	-0.016	0.113
	**QDT-T→SSE → RHD→ HRQOL**	0.030	0.003	0.011	0.059	0.041	0.002	0.016	0.080	0.010	0.272	-0.010	0.051
	**QDT-T→SSE →HRQOL**	0.050	0.009	0.013	0.098	0.052	0.066	-0.003	0.119	0.048	0.013	0.009	0.125
	**QDT-T → RHD→ HRQOL**	0.044	0.004	0.014	0.085	0.052	0.004	0.015	0.111	0.022	0.373	-0.032	0.090

Note: QDT: Quality of Discharge Teaching; QDT-R: QDT-Received content; QDT-T: QDT-Teaching skills and effects; SSE: Stoma Self-Efficacy; RHD: Readiness for Hospital Discharge; HRQOL: Health-Related Quality of Life

**Fig 4:**
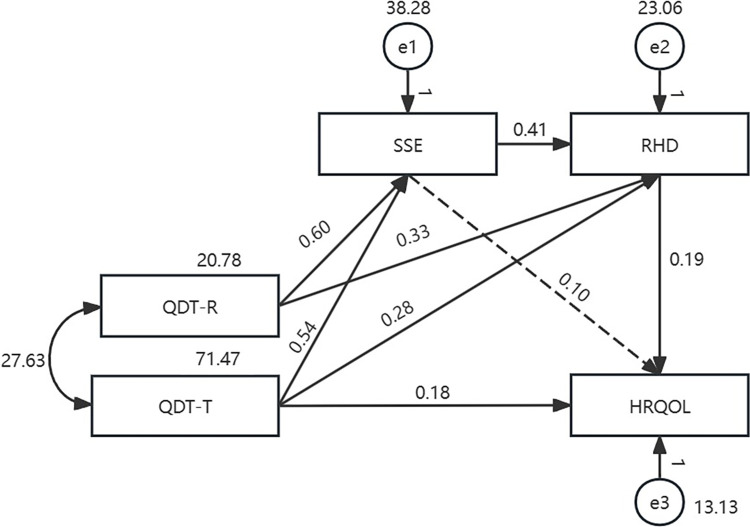
Path model with male colorectal cancer patients with temporary enterostomy (n = 144).

**Fig 5:**
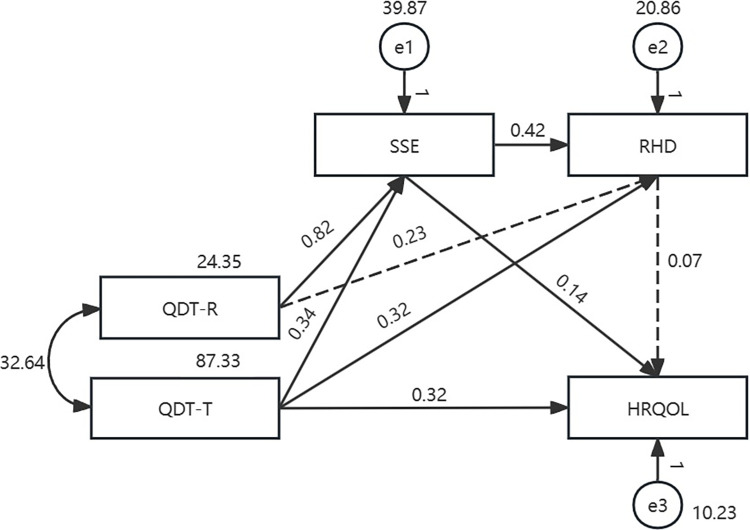
Path model with female colorectal cancer patients with temporary enterostomy (n = 77).

The two path submodels (model Ⅲ) using different sex-based groups are presented in Figs 4 and 5, and have a good model fit. The corresponding values are shown in Fig 4: χ2 = 1.457, df = 1, χ2/df = 1.457, GFI = 0.996, AGFI = 0.939, NFI = 0.997, CFI = 0.999, IFI = 0.999, TLI = 0.990, RMSEA = 0.057, and in Fig 5: χ2 = 1.573, df = 1, χ2/df = 1.573, GFI = 0.992, AGFI = 0.878, NFI = 0.995, CFI = 0.998, IFI = 0.998, TLI = 0.979, RMSEA = 0.087. Their path analysis results are shown in Table 3. As Fig 4 demonstrates, in the male patient group, the indirect effect accounted for 62.9% of the total effect (b = 0.312, *p*<0. 001, percentile 95% CI = [0.174, 0.468]), and among the indirect effect, the chain mediation of SSE to RHD was significant (b = 0.046, *p =* 0.004, percentile 95% CI = [0.011, 0.114]; b = 0.041, *P* = 0.002, percentile 95% CI = [0.016, 0.080]). In the female patient group, the chain mediation of SSE on RHD was not significant (b = 0.023, *p =* 0.309, percentile 95% CI = [-0.022, 0.121]; b = 0.010, *p* = 0.272, percentile 95% CI = [-0.010, 0.051]).

## Discussion

This is the first study to validate that SSE and RHD mediate the relationship between QDT-R and HRQOL, and QDT-T and HRQOL, and to certify that sex affects the interaction mechanism among colorectal cancer patients with temporary enterostomy. The findings of this study indicate that QDT has an indirect effect on post-discharge HRQOL through RHD. This study provides new evidence explaining how discharge planning affects HRQOL in colorectal cancer patients with stoma.

This study establishes a direct correlation between higher QDT-T scores and elevated levels of HRQOL but QDT-R does not among colorectal cancer patients with temporary enterostomy. Similarly, Zhang et al. [9] previously indicated that QDT-T affected the post-discharge HRQOL of patients with cataracts. This implies that implementing discharge planning with good teaching skills may improve the HRQOL of patients with stomas. In contrast, no significant direct relationship was identified between QDT-R and HRQOL in this study. Weiss et al. [14] previously explained that while it is beneficial to provide adequate teaching for discharge, less content is desirable for providing high quality QDT. An excess of teaching content may create an information overload and a psychological burden for patients. Thus, health care providers should pay more attention on how to select appropriate, high-quality discharge teaching content in clinical practices.

SSE was found to play a mediating role between QDT-R, QDT-T, and HRQOL among colorectal cancer patients with temporary enterostomy. Specifically, superior QDT forecasts heightened SSE, subsequently fostering a more satisfactory HRQOL. According to Meleis’ transitions theory [32], transition conditions can promote or hinder the transition process. Stoma formation may lead to various psychological problems such as depression, anxiety, body image change, low self-esteem, despair, and stigma [4]. These problems may hinder the patients’ transition from hospital to home [33]. SSE reflects the belief in one’s ability to master challenging demands by employing adaptive action [34]. It can stimulate patients to establish self-confidence and stoma self-care behavior, so as to obtain a successful transition [35, 36]. In clinical practices, health care providers could take SEE into account when delivering discharge planning, such as psychological support.

The findings of this study indicate that RHD also had a mediating effect on QDT and HRQOL, further confirming the results obtained in previous studies [10, 37]. Patients with a lack of or low level of readiness are much more likely to experience a difficult transition to home, and to fail to manage their home care situations [10]. Which may produce delayed discharge from the hospital to home or other settings, poor patient outcomes, distress, and increased medical costs [6]. RHD serves as an important risk indicator and trigger for discharge planning that can help to reduce the difficulties patients with stomas may encounter and increase their ability to cope with these problems. It implies that health care providers could enhance patients’ awareness of discharge preparation and identify high-risk stoma patients with delayed discharge and difficult discharge transition at an early stage.

This study also supports the chain-mediating role of SSE and RHD in the relationship between QDT-R and HRQOL, and between QDT-T and HRQOL, indicating that QDT can affect HRQOL via the path from SSE to RHD. Perceived SSE pertains explicitly to a patient’s coping confidence, ability, and adaptation to life [34, 38]. SSE can increase patients’ self-confidence and nursing ability after discharge to enhance their perception of RHD and jointly promote a more satisfactory HRQOL. This mediating relationship is clinically important for improving discharge quality. For example, it might help professional health providers to reach a better understanding of why stoma patients with the same QDT experience differences in HRQOL. Moreover, personnel can design SSE-enhancing and RHD-enhancing discharge planning for colorectal cancer patients with temporary enterostomies.

Sex has been well established as a predictor of HRQOL among colorectal cancer patients with stomas [21, 22]. This study aimed to identify sex-specific mediating HRQOL models. In the male patient model, the mediating effect of SSE on QDT and HRQOL was not significant, whereas the chain-mediating role of SSE via RHD was. In the female patient model, significant difference was identified between the mediating effect of SSE on QDT and HRQOL, while the chain-mediating role of SSE via RHD was not.

The different results corresponding to the different sex-based groups suggests that biological sex influences the interactive mechanisms between the two dimensions of QDT (QDT-R, and QDT-T), SSE, RHD, and HRQOL. Giordano et al. [39] reported that the variable of female sex was significantly associated with better self-care, resulting in a more practical basis for higher SSE. However, RHD in female patients with stomas may also be more seriously impaired by higher incidences of stigma, depression, and social isolation. Therefore, it is important to consider different approaches in discharge planning depending on the sex of the patient or patient group to enhance HRQOL for colorectal cancer patients with stomas.

## Limitations

This study has several limitations. First, HRQOL was measured only at one-month post-discharge, limiting our ability to examine HRQOL trajectories over time. Secondly, the sample was restricted to colostomy patients recruited from a single tertiary general hospital, introducing potential selection bias. Thirdly, unobservable selection bias between participants and non-participants cannot be ruled out. Finally, self-reported measurements may be subject to reporting bias, and other confounding factors were not considered. Future research should address these limitations.

## Conclusion

QDT-T can directly predict HRQOL, and QDT-T and QDT-R can predict HRQOL indirectly through three paths among colorectal cancer patients with stomas: (1) the mediating role of SSE, (2) the mediating role of RHD, and (3) the chain mediating role of SSE and RHD. The chain mediating effect of SSE and RHD is also significant in the male patient model. It provides a reference for health providers to better understand how QDT affect patients’ post-discharge HRQOL, and develop tailored discharge planning.

## Supporting information

S1 Checklist STROBE statement—Checklist of items that should be included in reports of *cross-sectional studies*. (DOCX)

S1 File Study data. (XLSX)
